# Bioequivalence Assessment of Sitagliptin/Metformin Extended-Release Fixed-Dose Combinations in Healthy Mexican Subjects Under Fasting and Fed Conditions

**DOI:** 10.3390/ph19101532

**Published:** 2026-09-27

**Authors:** Omar Emmanuel Hernández Piña, Porfirio de la Cruz Cruz, Erika Gabriela Guido Ávila, Alberto Martínez Muñoz, José Trinidad Pérez Urizar, Abraham Escobedo-Moratilla

**Affiliations:** 1 Asofarma de México S.A. de C.V., Av. Santa Fe 485, Lomas de Santa Fe, Contadero, Cuajimalpa de Morelos, Ciudad de Mexico C.P. 05348, Mexico; ophernandez@adium.com.mx (O.E.H.P.);; 2 Doctorado Institucional en Ingeniería y Ciencia de Materiales (DICIM), de la Universidad Autónoma de San Luis Potosí (UASLP), Av. Sierra Leona No. 550 Col. Lomas 2da. Sección, San Luis Potosí C.P. 78210, Mexico; 3 FS Scientia Pharma S.A. de C.V (Authorized Third Party), Av. Himno Nacional 1330, Jardín, Vista Hermosa, San Luis Potosí C.P. 78270, Mexico; 4 Facultad de Ciencias Químicas, de la Universidad Autónoma de San Luis Potosí, Av. Dr. Manuel Nava No. 6, Zona Universitaria, San Luis Potosí C.P. 78210, Mexico

**Keywords:** bioequivalence, pharmacokinetics, sitagliptin, metformin, fixed-dose combination, extended-release, food effect, Type 2 Diabetes

## Abstract

**Background/Objectives**: Management of Type 2 Diabetes (T2D) frequently requires combination therapy. Fixed-dose combinations (FDCs) of sitagliptin and extended-release (ER) metformin improve therapeutic adherence but pose biopharmaceutical challenges related to matrix robustness and food–drug interactions. This study evaluated the pharmacokinetics and bioequivalence of two generic sitagliptin/metformin ER FDCs (50/1000 mg and 100/1000 mg) versus reference products under fasting and fed conditions in healthy Mexican subjects. **Methods**: Four independent, randomized, open-label, two-period, single-dose crossover studies were conducted. We enrolled 184 healthy subjects (46 per study) with a 7-day washout period. Plasma concentrations of sitagliptin and metformin were quantified using a validated LC-MS/MS method. **Results**: The 90% confidence intervals for the geometric mean ratios (Test/Reference) of C_max_, AUC_0–t_, and AUC_0-**∞**_ for both analytes fell entirely within the 80.00–125.00% bioequivalence acceptance range across all studies. Under fed conditions, prolonged tmax and the absence of concentration spikes confirmed ER matrix preservation, with no evidence of food-induced dose dumping. The safety profile was highly favorable; all adverse events were mild or moderate and completely resolved without sequelae. **Conclusions**: The evaluated generic sitagliptin/metformin ER FDCs demonstrated pharmacokinetic bioequivalence to the reference products under fasting and fed conditions. These findings meet stringent regulatory requirements (NOM-177-SSA1-2013), support pharmacokinetic bioequivalence as the regulatory basis for safe therapeutic interchangeability, and provide robust, region-specific clinical data for the Latin American population. ClinicalTrials.gov identifiers: NCT07763899, NCT07763912, NCT07763951, and NCT07763964.

## 1. Introduction

Type 2 Diabetes (T2D) is a complex metabolic disorder characterized by peripheral insulin resistance and progressive pancreatic β-cell dysfunction, culminating in sustained hyperglycemia [1,2]. The global prevalence of T2D continues to escalate, representing a critical public health challenge. In Mexico, T2D is a leading cause of mortality and morbidity, with recent epidemiological data reporting a prevalence exceeding 18% in the adult population. This high disease burden highlights the urgent need for accessible, effective, and patient-compliant therapeutic strategies [3,4].

Current clinical guidelines recommend a multi-targeted pharmacological approach for T2D management to achieve optimal glycemic control and mitigate the risk of micro- and macrovascular complications [5,6]. Metformin, a biguanide that suppresses hepatic gluconeogenesis and improves peripheral insulin sensitivity, remains the foundational first-line therapy [7]. However, given the progressive nature of the disease, monotherapy often becomes insufficient, necessitating the addition of agents with complementary mechanisms of action. Sitagliptin, a highly selective dipeptidyl peptidase-4 (DPP-4) inhibitor, effectively addresses this need. By inhibiting the enzymatic degradation of endogenous incretin hormones—such as glucagon-like peptide-1 (GLP-1) and glucose-dependent insulinotropic polypeptide (GIP)—sitagliptin enhances glucose-dependent insulin secretion and suppresses inappropriate glucagon output [8,9]. The fixed-dose combination (FDC) of metformin and sitagliptin provides synergistic glycemic control while maintaining a risk of hypoglycemia [10].

To reduce pill burden and simplify treatment regimens, FDCs incorporating sitagliptin and extended-release (ER) metformin (such as the reference product Janumet^®^ XR) have been developed and are already widely prescribed in clinical practice [11]. Compared with the immediate-release formulation, ER metformin provides more sustained glycemic control, improved gastrointestinal tolerability, and enhanced patient adherence [12]. Nevertheless, developing these FDCs requires a sophisticated formulation strategy that integrates an immediate-release (IR) sitagliptin layer with an extended-release metformin core within a single dosage form while ensuring the distinct release profiles required for each API (Active Pharmaceutical Ingredient) [13,14]. From a biopharmaceutical perspective, sitagliptin and metformin possess distinct physicochemical and pharmacokinetic profiles. Sitagliptin, a Biopharmaceutics Classification System (BCS) Class I compound with high solubility and permeability [15], is rapidly absorbed with an absolute bioavailability of approximately 87%, and its systemic exposure is largely unaffected by a high-fat meal [16,17]. Its distribution is extensive (198 L at steady state) [17], with low plasma protein binding (38%). Although it undergoes minor hepatic metabolism, primarily mediated by cytochrome P450 3A4 (CYP3A4) and CYP2C8, it is predominantly eliminated unchanged via renal tubular secretion [18]. Although sitagliptin is a substrate of P-glycoprotein (P-gp) and several renal transporters, it maintains favorable oral absorption and systemic exposure.

Conversely, metformin is a Biopharmaceutics Classification System (BCS) Class III compound, characterized by high aqueous solubility but poor intestinal permeability [19]. Unlike sitagliptin, metformin does not undergo hepatic metabolism and exhibits negligible protein binding; it is excreted entirely unchanged in the urine. Metformin absorption is permeability-limited, dose-dependent (saturable), and relies on transporter-mediated intestinal uptake, primarily via organic cation transporters (OCT1) and the plasma membrane monoamine transporter (PMAT), which are predominantly expressed in the upper gastrointestinal tract [15]. Concomitant food intake delays gastric emptying, prolongs gastrointestinal transit times, and decreases metformin’s peak plasma concentration (C_max_) by approximately 40% and systemic exposure (AUC) by 25% [20]. Furthermore, because metformin ER formulations are designed to provide prolonged drug release and sustain drug delivery throughout the primary intestinal absorption window, they are clinically recommended to be taken with a meal. This recommendation optimizes bioavailability while minimizing the gastrointestinal intolerance commonly associated with biguanide therapy [21]. Consequently, rigorously evaluating the in vivo performance of these dual-release FDCs under both fasting and fed conditions is essential to satisfy regulatory expectations and ensure consistent drug release and predictable systemic drug levels in clinical practice [22].

Although the pharmacokinetics of sitagliptin and metformin have been characterized extensively in several regions, robust pharmacokinetic data specifically characterizing this ER combination in Latin American populations, particularly in Mexico, remain remarkably scarce. We selected the sitagliptin/metformin combination because it addresses multiple pathophysiological defects of T2D simultaneously, making it a cornerstone of modern glycemic management. Given the high prevalence of T2D in Mexico, locally generated biopharmaceutical evidence is crucial. Developing bioequivalent generic alternatives not only supports regulatory decision-making and reinforces confidence in therapeutic interchangeability within regional healthcare systems, but also addresses the socioeconomic need to improve patient access to these essential therapies [23].

Therefore, the present investigation aimed to characterize the pharmacokinetic profiles comprehensively and formally establish the bioequivalence of a new generic sitagliptin/metformin ER FDC in two dosage strengths (50/1000 mg and 100/1000 mg) against the innovator reference products. By conducting a robust clinical program comprising four independent randomized crossover trials in healthy Mexican volunteers under fasting and fed conditions, this study provides vital biopharmaceutical evidence supporting the pharmacokinetic bioequivalence of these formulations. It serves as a basis for their use in clinical practice.

For both strengths, the test and reference formulations exhibited highly comparable dissolution profiles, with calculated similarity factors (f_2_) of 73.97 and 89.52 for sitagliptin and metformin, respectively, in the 50/1000 mg strength, and 87.20 for metformin in the 100/1000 mg strength. For the 100/1000 mg sitagliptin strength, where the coefficient of variation exceeded 10% at the second sampling time for both products, similarity was assessed using the bootstrap f_2_ approach. The fifth percentile of the bootstrap f_2_ distribution (61.83) exceeded the acceptance criterion of 50, confirming similarity between the test and reference dissolution profiles.

Consistent with sitagliptin’s high aqueous solubility and BCS Class I classification, both formulations dissolved rapidly under the experimental conditions, with at least 85% of the labeled amount dissolved within 30 min for both strengths. In contrast, the metformin component exhibited the gradual dissolution profile characteristic of an extended-release formulation, reaching approximately 90% dissolved after 60–75 min.

## 2. Results

### 2.1. In Vitro Dissolution Profiles

Figure 1 and Figure 2 present the comparative dissolution profiles of sitagliptin and metformin from the test and reference extended-release fixed-dose combination tablets, respectively. Each figure includes the dissolution profiles for the two evaluated strengths (50/1000 mg and 100/1000 mg), corresponding to the same batches used in the in vivo bioequivalence studies.

### 2.2. Demographic Characteristics

A total of 184 healthy Mexican volunteers were enrolled across four independent, randomized, crossover bioequivalence studies (*n* = 46 per study). Table 1 summarizes baseline demographic characteristics, including gender distribution, age, weight, height, and body mass index (BMI).

The enrolled cohorts demonstrated a high degree of demographic homogeneity. Mean age was 34 ± 10 years, and mean BMI was well balanced across all four studies, ranging from 23.4 ± 2.0 kg/m^2^ to 24.3 ± 2.2 kg/m^2^. This uniformity in physical characteristics ensures baseline comparability across the study populations before drug administration.

Participant withdrawals and study flow are meticulously documented in the consolidated CONSORT flow diagram (Figure 3). Following the completion of the clinical phases, the final per-protocol (PP) populations—comprising subjects who completed both study periods and provided evaluable pharmacokinetic data without major protocol deviations—consisted of 44, 43, 44, and 44 participants for Studies 1 through 4, respectively. These PP populations were utilized for all subsequent pharmacokinetic and statistical analyses.

### 2.3. Pharmacokinetics and Bioequivalence

Following oral administration of the test and reference sitagliptin/metformin extended-release fixed-dose combinations (50/1000 mg and 100/1000 mg), the mean plasma concentration–time profiles for both analytes were virtually superimposable across all four independent studies under both fasting and fed conditions (Figure 4 and Figure 5).

A comprehensive summary of the primary and secondary pharmacokinetic parameters—including C_max_, t_max_, AUC_0–t_, AUC_0-∞_, and t_1/2_—for both sitagliptin and metformin is provided in Table 2. Across all evaluated dosage strengths and dietary states, the generic test formulations showed peak systemic exposure and total absorption extent highly comparable to those of the reference products. To formally assess bioequivalence, 90% confidence intervals (CIs) were calculated for the geometric mean ratios (Test/Reference) of the log-transformed parameters (C_max_, AUC_0–t_, and AUC_0-∞_).

As detailed in Table 3, all calculated metrics strictly met the predefined regulatory acceptance thresholds. For sitagliptin, the 90% CIs across the fasting and fed studies ranged from 89.35% to 106.72% for C_max_, from 94.27% to 102.58% for AUC_0–t_, and from 94.13% to 102.49% for AUC_0-∞_. Similarly, for metformin, the corresponding 90% CIs ranged from 94.09% to 116.05% for C_max_, from 94.92% to 112.80% for AUC_0–t_, and from 94.63% to 112.70% for AUC_0-∞_. The observed intra-subject variability (CV_intra_) for both analytes remained well within the expected limits for these molecules. Specifically, CV_intra_ values ranged from 7.0% to 21.7% for sitagliptin and from 7.4% to 18.2% for metformin across all PK parameters in the four cohorts. This moderate variability is consistent with the narrow confidence intervals and the robust statistical power (≥97%) observed in these trials.

Because all 90% CIs for both sitagliptin and metformin, across all dose strengths and prandial states, were entirely contained within the 80.00–125.00% acceptance margin, the bioequivalence of the test formulations was demonstrated. Furthermore, an analysis of variance (ANOVA) of the log-transformed pharmacokinetic data showed no statistically significant main effects (*p* > 0.05) for sequence, period, or treatment. The absence of significant sequence or period effects corroborates the validity of the two-way crossover design, confirms the adequacy of the 7-day washout period, and reinforces the robustness of the bioequivalence assessment.

### 2.4. Safety and Tolerability Analysis

The safety and tolerability profiles of the test and reference Sitagliptin/Metformin extended-release fixed-dose combinations were thoroughly monitored across all four clinical trials. Throughout the entire clinical development program, no serious adverse events (SAEs) were identified, and all affected participants achieved full clinical recovery without any residual sequelae.

Overall, 33 adverse events (AEs) were documented among the 184 enrolled healthy volunteers. A higher incidence of AEs was observed in the fed studies (22 events) than in the fasting cohorts (11 events). In the pooled fasting studies (50/1000 mg and 100/1000 mg), 11 AEs were registered. Of these, seven events (63.6%) occurred after the administration of the test formulation, while four events (36.4%) were associated with the reference product. The safety profile in the fasted state was characterized predominantly by mild-to-moderate headaches, with only isolated cases of dyspepsia and vomiting. Across the entire clinical development program, only one subject was withdrawn due to an AE (vomiting during the fasting trial).

Conversely, the pooled fed studies generated 22 AEs: 14 events (63.6%) linked to the test medication and eight events (36.4%) to the reference formulation. Clinical manifestations in the prandial state primarily consisted of headaches, followed by sporadic episodes of dyspepsia and loose stools. Most of these events were safely managed with standard pharmacological rescue therapies (e.g., ketorolac, metamizole, loperamide, or ondansetron) or resolved spontaneously under medical observation.

Overall, headache was the most prevalent adverse event, accounting for 24 of the 33 total documented episodes. A comprehensive breakdown detailing the specific nature, frequency, and distribution of all AEs across both dietary conditions is provided in Table 4.

As outlined in Table 4, the frequency and distribution of adverse events (AEs) were highly comparable between the test and reference interventions across all cohorts. Although the fed studies showed a marginally higher incidence of AEs than the fasting studies, we identified no clinically meaningful differences or systematic patterns between the generic and innovator products. All documented clinical manifestations were transient, required minimal intervention, and aligned with the established safety and pharmacological profiles of sitagliptin and metformin.

Unlike gender-restricted trials, the present clinical program enrolled healthy volunteers of both sexes. Across all evaluated dietary conditions and dose strengths, mild-to-moderate headaches dominated the tolerability profile. Gastrointestinal symptoms—such as dyspepsia, loose stools, nausea, and vomiting—occurred at considerably lower frequencies.

Participant discontinuations during the clinical execution phase were rigorously documented. Given the standard two-period, two-sequence (2 × 2) crossover design, intra-subject comparison is mathematically required to assess bioequivalence. Therefore, subjects who withdrew early lacked complete pharmacokinetic profiles for both the test and reference treatments, making this intra-subject comparison impossible. Consequently, they were excluded from the per-protocol population used for the final bioequivalence statistical analyses, although they were retained in the intention-to-treat population for the safety evaluation. The reasons for subject attrition across the four independent studies were as follows:

- 50/1000 mg Fasting Study: Two subjects were eliminated; one due to a reported adverse event (vomiting) during the therapeutic dosing interval of the first period, and one due to a positive urine pregnancy test before admission to the second period.
- 50/1000 mg Fed Study: Three subjects were discontinued following the voluntary withdrawal of their informed consent.
- 100/1000 mg Fasting Study: Two subjects were eliminated exclusively due to the voluntary withdrawal of their informed consent before dosing.
- 100/1000 mg Fed Study: Two subjects were withdrawn due to protocol non-compliance; one failed to consume the entirety of the standardized high-fat breakfast in the first period, and the other presented with a positive urine drug screen before the second period.

The only participant withdrawn due to an adverse event (vomiting) received close medical monitoring until complete symptom resolution was confirmed.

In strict adherence to the study protocols and the NOM-177-SSA1-2013 regulatory framework, all nine withdrawn participants were excluded from the pharmacokinetic and bioequivalence datasets, resulting in final per-protocol populations of 44, 43, 44, and 44 subjects for the respective studies.

## 3. Discussion

The clinical management of Type 2 Diabetes (T2D) frequently requires the concurrent use of pharmacological agents with complementary mechanisms of action to achieve and maintain stringent glycemic targets [24]. The combination of sitagliptin, a dipeptidyl peptidase-4 (DPP-4) inhibitor, and metformin, a biguanide, addresses multiple pathophysiological defects of T2DM by simultaneously enhancing incretin levels and improving hepatic insulin sensitivity [25]. Furthermore, the single-pill extended-release (ER) formulation significantly reduces pill burden. It simplifies the daily dosing regimen compared with taking individual immediate-release tablets, a theoretical benefit expected to improve patient adherence to chronic treatment [26,27]. Given the therapeutic and practical advantages of this fixed-dose combination, the bioequivalence demonstrated in the present work strongly supports the pharmacokinetic bioequivalence of the evaluated generic formulations and provides the regulatory foundation for clinical interchangeability. Across the two dosage strengths (50/1000 mg and 100/1000 mg) rigorously evaluated under both fasting and fed conditions in healthy Mexican subjects, the primary pharmacokinetic parameters, C_max_, AUC_0–t_, and AUC_0-∞_, for both analytes consistently met the predefined regulatory acceptance criteria of 80.00% to 125.00%.

The highly comparable dissolution behavior observed for both active pharmaceutical ingredients provides additional pharmaceutical support for the bioequivalence demonstrated in vivo. Although the present study was not designed to establish or validate an in vitro–in vivo correlation (IVIVC), the dissolution methodology was selected in accordance with product-specific regulatory recommendations, and previous investigations of sitagliptin/metformin extended-release formulations support its scientific rationale. Boddu et al. developed and validated a Level A IVIVC model for a fixed-dose combination containing immediate-release sitagliptin and extended-release metformin under both fasted and fed conditions [28]. Using dissolution methods designed to represent physiologically relevant gastrointestinal environments, specifically USP Apparatus I (basket) operated at 100 rpm with dissolution media at pH 6.8 (fasted) and pH 5.5 (fed), they demonstrated strong linear relationships between the fraction dissolved and the fraction absorbed for both active ingredients, with correlation coefficients exceeding 0.90 and internal and external prediction errors below 10%, thereby satisfying regulatory validation criteria. Although these experimental conditions differed from the product-specific dissolution methodology employed in the present study, both approaches were designed to characterize drug release under physiologically relevant gastrointestinal conditions. These findings demonstrate that appropriately selected dissolution methodologies may establish meaningful relationships between in vitro drug release and the corresponding in vivo performance of sitagliptin/metformin formulations [28].

Interpreting dissolution data is particularly relevant for extended-release metformin formulations because the drug’s biopharmaceutical characteristics influence the relationship between drug release and systemic exposure. Balan et al. evaluated extended-release metformin using USP Apparatus II (paddle) operated at 100 rpm in phosphate buffer (pH 6.8), representing intestinal conditions, and reported that in vivo input from modified-release metformin formulations is likely to be predominantly controlled by absorption, or by a combination of absorption and dissolution, reflecting the drug’s low permeability and site-dependent intestinal absorption [29]. Although experimental conditions and formulation characteristics differed from those of the present study, these findings indicate that the in vivo performance of extended-release metformin should be interpreted as the result of an interplay between formulation-controlled drug release and intestinal absorption rather than either process alone.

In this context, the close agreement between the dissolution behavior and the bioequivalence observed in the present study is consistent with previously reported biopharmaceutical characteristics of sitagliptin/metformin extended-release formulations. Although no IVIVC was established, the use of a dissolution methodology supported by regulatory recommendations and previous IVIVC investigations, together with the demonstrated in vivo bioequivalence, provides complementary pharmaceutical evidence supporting the in vitro release performance and pharmaceutical comparability of the evaluated extended-release sitagliptin/metformin fixed-dose combination tablets.

These pharmacokinetic findings are particularly noteworthy when evaluated through the lens of the Biopharmaceutics Classification System (BCS) and the drugs’ respective absorption mechanisms. Metformin is a well-characterized BCS Class III compound, possessing high aqueous solubility but low intestinal permeability [30]. Because its absorption is highly dependent on saturable active transporters (such as OCT1 and PMAT), variations in formulation excipients could theoretically alter its intestinal uptake [31]. Similarly, sitagliptin interacts with specific efflux and uptake transporters, acting as a substrate for P-glycoprotein (P-gp) and the human organic anion transporter-3 (hOAT-3) [32]. Because absorption of such compounds can be sensitive to formulation-related factors [33], we hypothesize that the closely comparable pharmacokinetic profiles observed between the generic and innovator products suggest that formulation differences did not result in clinically meaningful changes in systemic exposure to either active ingredient.

A primary regulatory and clinical concern when evaluating ER formulations is the potential for food-induced dose dumping. In this phenomenon, the physicochemical stress of a high-fat, high-calorie meal prematurely compromises the polymer matrix, causing rapid, unintended release of the entire drug payload [34,35]. The absence of an abrupt peak-exposure spike, coupled with a prolonged t_max_ under fed conditions, supports the generic matrix’s structural robustness [36]. The exploratory food-effect assessment yielded further insight into these observations. While food intake increased the systemic exposure of metformin in both the test and reference formulations—as reflected by higher C_max_ and AUC values—these findings must be interpreted strictly within the context of the study design. Because the fasting and fed investigations were conducted as independent bioequivalence trials in parallel cohorts rather than as a dedicated, single-population crossover food-effect study, the cross-study fed-to-fasting comparisons remain purely descriptive. Therefore, we did not apply formal inferential statistical testing or predefined regulatory equivalence margins. Importantly, despite the observed food-related increase in overall systemic exposure, the concomitant intake of a high-fat meal was consistently associated with a prolonged rather than shortened t_max_ for both active ingredients. Taken together, these findings support the hypothesis that the extended-release characteristics of the generic formulation were maintained under fed conditions, a behavior fundamentally inconsistent with food-induced dose dumping [37].

Regarding metabolic disposition, the observed pharmacokinetic parity provides a basis to discuss the low susceptibility of this fixed-dose combination to inter-ethnic genetic variation. Metformin undergoes virtually no hepatic metabolism and is excreted entirely unchanged via tubular secretion [38]. Concurrently, sitagliptin is predominantly eliminated by the kidneys, with renal clearance accounting for approximately 70% of its total plasma clearance [39]. Although sitagliptin undergoes minor hepatic biotransformation, mediated primarily by CYP3A4 and CYP2C8, the cytochrome P450 (CYP450) enzyme system plays a negligible overall role in its systemic clearance [40]. Consequently, we hypothesize that the contribution of CYP-mediated metabolism to total sitagliptin clearance is limited, suggesting that CYP polymorphisms are unlikely to be major determinants of systemic exposure.

To further contextualize these physiological premises, our cross-regional data comparisons corroborate these pharmacokinetic findings by highlighting the behavior of both active ingredients across diverse global demographics. Regarding the DPP-4 inhibitor, the fasting maximum plasma concentrations (C_max_) observed in our Mexican cohort for sitagliptin 50 mg (approximately 212–220 ng/mL) and 100 mg (approximately 480–500 ng/mL) align closely with the exposure levels reported in an open-label, two-period crossover study evaluating a 50/850 mg formulation in healthy Chinese subjects [16], where the fasting sitagliptin C_max_ ranged from 162.93 to 182.37 ng/mL. Furthermore, a dedicated absolute bioavailability study of a 100 mg dose of sitagliptin [17]—which demonstrated an 87% absolute oral bioavailability—established that a high-fat meal does not significantly alter its total systemic absorption, yielding geometric mean ratios (fed/fasting) of 1.03 for AUC and 0.94 for C_max_. This behavior is highly consistent with the stable sitagliptin AUC ratios and the lack of food-induced absorption impairment observed in our fed cohorts. For the biguanide component, the fasting C_max_ for the metformin 1000 mg dose in our study (1210–1502 ng/mL) is pharmacokinetically comparable to the levels reported by Migoya et al. [41] in a predominantly Hispanic-American population (94% of the cohort), where a 50/1000 mg fixed-dose combination yielded a metformin C_max_ ranging from 1850 to 1870 ng/mL. Additionally, our exposure metrics are consistent with those observed in a bioequivalence study involving healthy Nigerian volunteers [42], which reported a C_max_ of 0.43 μg/mL (430 ng/mL) following a single 500 mg immediate-release dose. It is also noteworthy that, while Shi et al. [16] reported a significant decrease in metformin exposure (both C_max_ and AUC) under fed conditions for a 50/850 mg formulation in Chinese subjects, our extended-release formulation demonstrated a well-documented increase in the extent of metformin absorption, along with a prolonged t_max_. These detailed cross-regional comparisons support the hypothesis that, despite minor inter-ethnic variations in absolute exposure and the expected pharmacokinetic differences inherent to immediate versus extended-release matrices, the overall disposition, dose-proportionality, and safety profiles of both drugs remain highly predictable across diverse populations.

The safety and tolerability findings offer robust clinical support for the pharmacokinetic observations, confirming the highly favorable performance of the generic ER formulations. However, in broader clinical practice, the combination of sitagliptin and metformin presents specific challenges and limitations. Metformin therapy is frequently associated with dose-dependent gastrointestinal intolerance (e.g., diarrhea, nausea, and dyspepsia). It carries a rare but severe risk of lactic acidosis, necessitating cautious use and dose adjustments in patients with renal impairment. Conversely, while sitagliptin is generally well-tolerated, it has been linked to rare adverse events such as acute pancreatitis, severe disabling joint pain, and hypersensitivity reactions. Furthermore, a fixed-dose combination inherently limits dosing flexibility, making independent titration of either active ingredient impossible if a patient experiences specific adverse effects or changes in renal function. In the present study, while gastrointestinal events (such as nausea, dyspepsia, or abdominal pain) are classically associated with metformin administration [43], the overall incidence across these healthy cohorts remained low, and all documented events were strictly classified as mild to moderate. These observations are consistent with previously published bioequivalence studies of sitagliptin/metformin fixed-dose combinations, in which headache and mild gastrointestinal events represented the most frequently reported adverse events. The favorable tolerability profile observed in the present studies is consistent with controlled drug release and the absence of excessive systemic exposure [44,45]. Furthermore, participant discontinuations across all four studies were predominantly attributable to voluntary withdrawal of consent or protocol non-compliance, rather than severe drug-related adverse events. The rigorous crossover design of our studies effectively controlled for inter-subject physiological variability, suggesting that the demonstrated bioequivalence and similar safety profiles reflect pharmaceutical formulation design rather than individual metabolic phenotypes.

Like most standard bioequivalence assessments, these trials were conducted under single-dose conditions in a healthy, relatively young demographic. While including patients with Type 2 Diabetes (T2D) is essential for evaluating clinical efficacy, this study was specifically designed to assess pharmacokinetic bioequivalence. In accordance with major regulatory guidelines, bioequivalence trials are preferentially conducted in healthy volunteers to minimize intra- and inter-subject pharmacokinetic variability caused by disease progression, altered renal function, or concomitant medications, thereby isolating formulation performance. While this represents the rigorous regulatory standard for demonstrating formulation equivalence, it may not fully capture steady-state pharmacokinetics or the long-term tolerability profile in T2DM patients with varying degrees of renal impairment, altered gastric motility, or polypharmacy. Additionally, assumptions regarding transporter interactions and long-term compliance remain exploratory. Nevertheless, the rigorous design effectively minimized inter-subject variability, isolating the formulation effect as the primary variable.

While the present study robustly establishes pharmacokinetic bioequivalence, several avenues for future research remain available to further characterize these formulations’ clinical profile in real-world settings. The innovator manufacturer originally established the efficacy, safety, dose optimization, and overall benefit–risk profile of the sitagliptin/metformin extended-release combination through a comprehensive clinical development program. Consequently, generic development relies primarily on demonstrating pharmaceutical equivalence and bioequivalence, avoiding unnecessary duplication of large clinical efficacy and safety trials while preserving the established evidence base supporting therapeutic use. This scientifically and ethically justified regulatory framework also improves affordability and broadens access to effective therapies for patients with T2D. Within this context, future studies should evaluate these FDCs in patients with T2D, particularly those with varying degrees of renal function, to confirm broader clinical applicability. Multiple-dose and steady-state studies would further characterize long-term pharmacokinetics, safety, tolerability, and patient adherence. Additionally, while our parallel-cohort findings suggest matrix preservation under fed conditions, a dedicated randomized crossover food-effect study within the same participant cohort would allow a more precise quantification of food-related pharmacokinetic changes. Likewise, establishing a formal in vitro–in vivo correlation (IVIVC) could provide valuable mechanistic insights linking dissolution and release characteristics with drug absorption. Finally, expanding population diversity by including different ethnic groups, wider age ranges, and patients with comorbidities or concomitant medications will help support the generalizability and safe interchangeability of these generic ER formulations in routine clinical practice.

## 4. Materials and Methods

### 4.1. Drug Products

For the clinical assessments, the experimental test treatments comprised generic Sitagliptin/Metformin extended-release tablets at two dose strengths (50/1000 mg and 100/1000 mg), produced by Farmacéutica Paraguaya, S.A. (Asunción, Paraguay). These were systematically evaluated against the corresponding innovator counterparts, Janumet^®^ XR (Sitagliptin/Metformin) extended-release tablets of the same strengths. The reference formulations were manufactured by MSD International GmbH (Puerto Rico Branch) LLC (Las Piedras, PR, USA) and legally sourced from the authorized Mexican supply chain via Merck Sharp & Dohme Comercializadora, S. de R.L. de C.V. (Mexico City, Mexico).

Before the initiation of the dosing phases, the specific batch designations (Test: 75625 for the 50/1000 mg strength and batch 75284 for the 100/1000 mg strength; Reference: batch B120119 for the 50/1000 mg strength and B120346 for the 100/1000 mg strength) and expiry dates for all investigational products were subjected to strict verification. Analytical quality control reviews ensured that both the generic and brand-name medications complied with the rigorous acceptance criteria established by the Mexican regulatory standard NOM-177-SSA1-2013 for bioequivalence trials [46]. Notably, assay differences between the corresponding test and reference batches were below 5%, and dosage unit uniformity was satisfactory.

To ensure product integrity throughout the studies, all pharmaceutical supplies were stored in a restricted-access clinical pharmacy under the direct custody of the designated pharmacist. Storage conditions were continuously monitored in accordance with Good Clinical Practice (GCP) principles and the manufacturers’ recommended storage conditions until dosing.

### 4.2. In Vitro Dissolution Studies

Comparative in vitro dissolution studies were performed using the same test and reference batches evaluated in the in vivo bioequivalence studies to characterize the release performance of the formulations under the dissolution conditions recommended by the FDA Dissolution Methods Database [47]. The formulations evaluated comprised the 50/1000 mg strength (test batch 75625 and reference batch B120119) and the 100/1000 mg strength (test batch 75284 and reference batch B120346).

Dissolution testing was conducted using USP Apparatus II (paddle) with 900 mL of pH 6.8 maintained at 37.0 ± 0.5 °C and a rotation speed of 75 rpm. Cage-type sinkers were used throughout the study to ensure proper tablet positioning during dissolution. Twelve dosage units of each test and reference formulation were evaluated. Samples were collected at predetermined time points, filtered through 0.45 μm Nylon Titan^®^ membrane filters, and analyzed without replacement of the dissolution medium.

The amounts of dissolved sitagliptin and metformin was quantified by high-performance liquid chromatography coupled with UV detection (HPLC–UV) using a Phenomenex^®^ Luna SCX column (50 × 4.6 mm, 5 μm). Chromatographic separation was performed under isocratic conditions using a mobile phase consisting of potassium dihydrogen phosphate buffer (pH 3.5) and acetonitrile (75:25, v/v), at a flow rate of 2.0 mL/min. The column temperature was maintained at 30 °C, the injection volume was 5 μL, and detection was carried out at 205 nm.

Comparative dissolution profile analyses were performed in accordance with the criteria established in NOM-177-SSA1-2013 for interchangeability assessments [46]. Similarity between the test and reference formulations was primarily evaluated using the similarity factor (f_2_), calculated with the DDSolver add-in for Microsoft Excel [48]. Dissolution profiles were considered similar when the f_2_ value was ≥50 [49]. When the coefficient of variation exceeded 20% at the first sampling time or 10% at any subsequent sampling time, dissolution profile similarity was assessed using the bootstrap f_2_ approach (5000 bootstrap resamples), following current regulatory recommendations [46,50]. Under this approach, similarity was concluded when the fifth percentile of the bootstrap f_2_ distribution was ≥50 [51].

### 4.3. Study Design

Before the clinical phase began, the clinical protocols and informed consent forms received full approval from the independent Ethics in Research Committee (CEI), the Institutional Research Committee (IRC), and the Mexican health authority (COFEPRIS). The four clinical trials were executed in strict compliance with the ethical tenets of the Declaration of Helsinki (including the 75th WMA General Assembly revisions, 2024) [52], the International Council for Harmonization (ICH) Good Clinical Practice (GCP) guidelines (E6 R3, 2025) [53], and the Mexican regulatory norm NOM-177-SSA1-2013 [46].

To characterize the pharmacokinetic behavior of the Sitagliptin/Metformin extended-release tablets (50/1000 mg and 100/1000 mg), we conducted four independent, single-center, single-dose, open-label, randomized, two-period, two-sequence (2 × 2) crossover trials under both fasting and fed conditions. All clinical phases were conducted at the facilities of Axis Clinicals Latina, S.A. de C.V. (Mexico City, Mexico).

A total of 46 healthy volunteers were randomized in each study. During confinement, all participants received standardized meals providing approximately 2500 kcal/day. For both fasting and fed studies, subjects consumed a standardized dinner on the evening before dosing and completed a supervised overnight fast of at least 10 h. In the fasting cohorts, study medication was administered under fasting conditions, and a standardized breakfast was served approximately 4 h post-dose, followed by scheduled meals and snacks. In the fed cohorts, subjects received a standardized high-fat, high-calorie breakfast 30 min before dosing. This meal provided approximately 969 kcal, including 558 kcal from fat, 261 kcal from carbohydrates, and 150 kcal from protein, corresponding to approximately 58%, 27%, and 15% of total caloric intake, respectively. All subsequent meals and snacks were standardized and equivalent to those administered in the fasting studies. For each study period, volunteers were admitted to the clinical unit approximately 12 h before dosing and remained confined until the 24 h post-administration blood draw was completed. Subsequent pharmacokinetic samples were collected during scheduled ambulatory visits at 36.00, 48.00, and 72.00 h post-dose. To ensure complete drug clearance and avoid potential carryover effects, a strict 7-day washout interval was maintained between the two treatment periods. Throughout the confinement and ambulatory monitoring phases, the clinical staff continuously assessed participants’ vital signs and meticulously documented any emergent adverse events, including their severity, duration, and causal relationship to the investigational products.

### 4.4. Study Population

Enrollment in the four clinical trials was completely voluntary. Before any study-related screening evaluations were conducted, all prospective subjects provided written informed consent. The pooled study population consisted of healthy Mexican adult volunteers of both sexes, aged 18 to 55 years, with a body mass index (BMI) ranging from 18.0 to 27.0 kg/m^2^. These demographic parameters align with the regulatory stipulations dictated by the NOM-177-SSA1-2013 guideline [46].

To ascertain optimal health conditions, participants underwent rigorous clinical and diagnostic screening, with laboratory and 12-lead electrocardiogram (ECG) results remaining valid for up to 3 months before study initiation. This thorough evaluation encompassed a comprehensive medical history, physical examination, and standard laboratory panels (blood chemistry, hematology, and urinalysis). Additionally, biosafety assessments included serological screening for HIV, hepatitis B and C, and VDRL, alongside testing for drugs of abuse and pregnancy (in female subjects). At the time of admission to the clinical facility for each dosing period, eligibility was systematically reconfirmed through rapid urine drug tests and pregnancy screenings.

Principal exclusion criteria applied across all study arms included any clinically significant history of cardiovascular, hepatic, renal, endocrine, respiratory, or gastrointestinal disorders that could compromise subject safety or alter the pharmacokinetic disposition of Sitagliptin/Metformin. Further grounds for exclusion included a recent history of substance or alcohol abuse, blood donation exceeding 450 mL within 60 days, and participation in any other clinical trial within the preceding three months. Volunteers were also prohibited from utilizing any pharmacological treatments within 14 days (or seven half-lives) before dosing, and female participants were expressly forbidden from using hormonal contraceptives as a birth control method.

### 4.5. Sample Size

In accordance with NOM-177-SSA1-2013 regulatory guidelines [46], the sample size was determined based on the pharmacokinetic parameter with the highest intra-subject coefficient of variation (CV_intra_) between the two active pharmaceutical ingredients. Based on the international literature [54] and internal clinical data (ACL22-PC019, 2023), a maximum CV_intra_ of 25.2% was identified for the C_max_ of Sitagliptin in extended-release formulations. In contrast, the variability for Metformin was documented to be slightly lower, reaching up to 24.2% ([55] and internal study ACL18-PC039). Consequently, we used the 25.2% CVintra as the basis for the sample size calculation.

Sample size estimates were generated using the Bear package in the R statistical computing environment (version 4.1.2 [56]), applying accepted formulas for crossover designs [57]. The calculations were based on a predefined framework: a Type I error (α) of 0.05, a target statistical power of 90% (Type II error, β = 0.10), an expected test-to-reference geometric mean ratio of 95%, and an equivalence margin of 80.00% to 125.00% (corresponding to a ±20% equivalence margin). The target statistical power was set at 90%, exceeding the minimum requirement of 80%, to ensure adequate power for the bioequivalence evaluation.

Based on these statistical parameters, a minimum cohort of 38 evaluable subjects was required to achieve the target statistical power for the bioequivalence assessment of both Sitagliptin and Metformin. To account for potential participant attrition, adverse events, or voluntary withdrawals, we included 8 additional subjects in the planned enrollment. This approach was intended to ensure that the required number of evaluable participants remained available for analysis; the adjustment was designed not to exceed a 20% maximum surplus. Therefore, the final sample size was established at 46 randomized subjects per study.

### 4.6. Pharmacokinetic Assessment and Analytical Method

To characterize the pharmacokinetic profiles of sitagliptin and metformin, sequential venous blood samples (approximately 6 mL each) were collected into Vacutainer^®^ tubes containing K_2_EDTA as the anticoagulant. For each study period, blood samples were obtained before drug administration (0.00 h) and at 0.25, 0.50, 0.75, 1.00, 1.25, 1.50, 1.75, 2.00, 2.50, 3.00, 3.50, 4.00, 4.50, 5.00, 5.50, 6.00, 7.00, 8.00, 10.00, 12.00, 16.00, 24.00, 36.00, 48.00, and 72.00 h after dosing. Following collection, blood samples were centrifuged to separate plasma, which was transferred into pre-labeled cryogenic tubes and stored at −70 ± 15 °C until bioanalytical determination.

Plasma concentrations of sitagliptin and metformin were simultaneously quantified using a validated liquid chromatography–tandem mass spectrometry (LC–MS/MS) method developed and validated by the bioanalytical laboratory of Axis Clinicals Latina, S.A. de C.V. (Mexico City, Mexico). Method validation was performed in accordance with Good Laboratory Practice (GLP), applicable Mexican regulatory (NOM-177-SSA1-2013) requirements, and internationally recognized bioanalytical method validation guidelines.

Sample preparation was performed by solid-phase extraction (SPE). Briefly, 300 µL of human plasma was transferred to polypropylene tubes and mixed with 50 µL of an internal standard solution containing sitagliptin-d4 and metformin-d6; then, 400 µL of a pretreatment solution was added. After vortex mixing, the samples were loaded onto preconditioned Strata-X SPE cartridges previously activated with methanol and equilibrated with deionized water. The cartridges were subsequently washed with deionized water and 5% methanol to remove endogenous matrix components before analyte elution with the mobile phase. The resulting extracts were transferred to autosampler vials equipped with glass inserts, and 10 µL was injected into the LC–MS/MS system.

Chromatographic separation was performed using a Prominence LC-20AD/SIL-20AC high-performance liquid chromatography system (Shimadzu, Kyoto, Japan) equipped with a Phenomenex Kinetex^®^ C_8_ column (150 × 4.6 mm, 5 µm, 100 Å). The mobile phase consisted of methanol, acetonitrile, and 25 mM ammonium formate (70:5:25, *v*/*v*/*v*), delivered at a flow rate of 0.800 mL/min. The autosampler and column temperatures were maintained at 5 °C and 40 °C, respectively. A 10 µL aliquot was injected for each analysis, and the chromatographic run time was 3.5 min.

Mass spectrometric detection was performed using an API 4000 or API 4500 triple quadrupole mass spectrometer (AB Sciex, Singapore) equipped with a TurboIonSpray interface operating in positive electrospray ionization mode (ESI+). Quantification was achieved by multiple reaction monitoring (MRM) using the following precursor-to-product ion transitions: *m*/*z* 408.4 → 235.2 for sitagliptin, *m*/*z* 130.2 → 71.1 for metformin, *m*/*z* 412.3 → 239.2 for sitagliptin-d4, and *m*/*z* 136.2 → 77.1 for metformin-d6.

Calibration curves covered concentration ranges of 2.00–600.21 ng/mL for sitagliptin and 10.0–3001.0 ng/mL for metformin. Quality control (QC) samples were prepared at four concentration levels for each analyte: 5.98, 249.14, and 457.97 ng/mL for sitagliptin, and 29.9, 1247.0, and 2280.0 ng/mL for metformin. Quantification was performed using Analyst^®^ software (version 1.6.2) by linear regression of analyte-to-internal-standard peak area ratios against nominal concentrations using a 1/x^2^ weighted calibration model.

The validated bioanalytical method demonstrated acceptable selectivity, linearity, accuracy, precision, recovery, matrix effect, sensitivity, and stability throughout the validated concentration ranges. A comprehensive summary of the bioanalytical method validation, including calibration performance and all validation parameters, is provided in the Supplementary Materials.

### 4.7. Tolerability and Safety Assessments

The safety and tolerability of the Sitagliptin/Metformin extended-release fixed-dose combinations were meticulously evaluated throughout the confinement and ambulatory phases of all four clinical trials. Participants underwent thorough clinical examinations, including detailed physical assessments by qualified medical personnel during the initial screening, upon admission to the clinical facility for each period, and immediately before their final discharge.

To ensure participant safety and assess potential hemodynamic or systemic fluctuations, vital signs—including systolic and diastolic blood pressure, heart rate, and body temperature—were systematically monitored. These parameters were recorded at baseline (approximately 05:00 h, before dosing) and at predetermined post-dose intervals throughout the inpatient period (mid-morning, evening, and the following morning before discharge) across both the fasting and fed cohorts.

The incidence, severity, and causality of any emergent adverse events (AEs) were rigorously tracked and documented in strict accordance with the Mexican pharmacovigilance standard NOM-220-SSA1-2016 [58]. AEs were captured through spontaneous participant reporting and active, continuous surveillance by the clinical investigating team. Each documented event was formally classified by severity (mild, moderate, or severe), overall duration, need for clinical or pharmacological intervention, and ultimate resolution. This rigorous methodological approach established a comprehensive and reliable safety profile for the evaluated test and reference formulations.

### 4.8. Statistical Analysis

Baseline demographic and clinical features for all four study cohorts were summarized using standard descriptive statistics. Safety and tolerability evaluations were conducted in the intention-to-treat (ITT) population, comprising all 184 randomized volunteers (46 per study) who received at least one dose of the investigational products. In contrast, pharmacokinetic (PK) and bioequivalence statistical inferences were strictly based on the per-protocol (PP) datasets. Because the statistical framework for a standard 2 × 2 crossover design requires intra-subject comparisons between the test and reference formulations, the PP population was strictly restricted to subjects who completed both study periods and provided full, evaluable concentration–time profiles for both treatments without critical protocol deviations. Subjects who dropped out prematurely could not contribute to the intra-subject variance evaluation and were therefore excluded from this specific analysis. After accounting for clinical withdrawals, the final evaluable samples comprised 44, 43, 44, and 44 subjects in studies 1, 2, 3, and 4, respectively.

The primary PK parameters established to definitively ascertain bioequivalence were the maximum observed plasma concentration (C_max_), the area under the concentration–time curve from time zero to the last measurable sampling point (AUC_0–t_), and the area under the curve extrapolated to infinity (AUC_0-∞_). Secondary kinetic variables evaluated included the time to reach maximum concentration (t_max_), the elimination rate constant (K_e_), the terminal half-life (t_1/2_), and the mean residence time (TMR).

All PK parameters were computed via non-compartmental analysis (NCA) utilizing Phoenix^®^ WinNonlin^®^ software, version 8.7 (Certara L.P., Radnor, NJ, USA). While C_max_ and t_max_ were derived directly from the empirical plasma concentration–time profiles, AUC_0–t_ was determined using the linear trapezoidal integration method. AUC_0-∞_ was calculated as the sum of AUC_0–t_ and the extrapolated terminal area (C_last_/K_e_), where the terminal elimination rate constant (K_e_) was estimated by linear regression of the terminal log-linear portion of the concentration–time profile.

To evaluate bioequivalence, Schuirmann’s two one-sided t-tests (TOST) were performed on the log-transformed primary metrics (C_max_, AUC_0–t_, and AUC_0-∞_). We performed an analysis of variance (ANOVA) within a Generalized Linear Model (GLM) to detect statistically significant variations attributable to the administration sequence, subjects nested within sequence, clinical period, and treatment formulation. In strict adherence to the Mexican regulatory guideline NOM-177-SSA1-2013 [46], 90% confidence intervals (CIs) were generated for the geometric mean ratios (Test/Reference). Bioequivalence was formally declared if the resultant 90% CIs for C_max_, AUC_0–t_, and AUC_0-∞_ were completely encompassed within the predefined regulatory acceptance margins of 80.00% to 125.00%.

## 5. Conclusions

In conclusion, the generic sitagliptin/metformin extended-release (ER) fixed-dose combinations (50/1000 mg and 100/1000 mg) successfully demonstrated bioequivalence to the reference innovator products under both fasting and fed conditions in healthy Mexican volunteers. Across all evaluated clinical cohorts, the primary pharmacokinetic parameters (C_max_, AUC_0–t_, and AUC_0-∞_) consistently fell within the regulatory acceptance limits of 80.00% to 125.00%. Furthermore, the comparable pharmacokinetic profiles suggest that formulation differences do not result in clinically meaningful changes in the absorption or systemic exposure of either active ingredient.

The exploratory food-effect assessment further supported the formulation’s structural robustness. The prolonged t_max_ and the absence of abrupt concentration spikes under fed conditions support the ER matrix’s controlled-release characteristics. These findings provide no pharmacokinetic evidence of food-induced dose dumping, supporting a safe, controlled drug absorption profile across different prandial states. Additionally, cross-regional comparisons with international data underscore a predictable disposition profile, suggesting generally consistent pharmacokinetic behavior across populations evaluated in previous studies.

Methodologically, the rigorous crossover design and enrollment of an optimal sample size (yielding exceptional statistical power) effectively mitigated anticipated intra-subject variability. This robust execution not only strengthens the reliability of the present findings but also establishes a solid benchmark for future bioequivalence evaluations of complex, highly soluble, and low-permeability compounds.

Clinical observations closely corroborated the pharmacokinetic data, revealing a highly favorable safety and tolerability profile. All documented adverse events were mild, transient, and resolved without sequelae. Most importantly, the low incidence of gastrointestinal adverse events—which are classically associated with immediate-release metformin—demonstrates that the ER mechanism effectively mitigates peak-related systemic toxicity. We hypothesize that this improved tolerability profile may theoretically promote long-term therapeutic adherence in clinical practice.

Altogether, these findings fulfill the strict bioequivalence and safety requirements mandated by the Mexican regulatory authority (COFEPRIS) under the NOM-177-SSA1-2013 framework. The comprehensive pharmacokinetic, biopharmaceutical, and clinical evidence strongly supports the pharmacokinetic bioequivalence of these generic ER formulations, supporting their safe interchangeability under regulatory standards and establishing them as reliable, efficacious, and accessible options to help alleviate the economic burden of managing Type 2 Diabetes in Latin American populations.

## Figures and Tables

**Figure 1:**
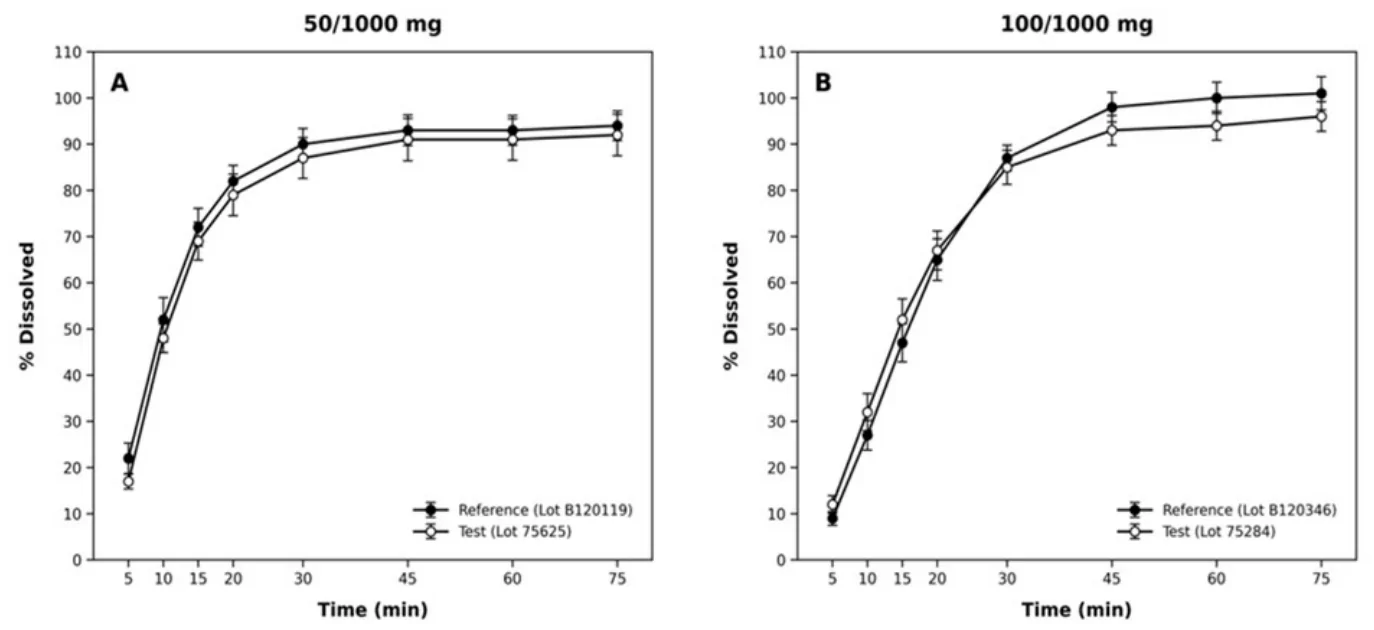
Mean comparative dissolution profiles of sitagliptin from test (white circles) and reference (black circles) sitagliptin/metformin extended-release fixed-dose combination tablets. The panels represent: (**A**) 50/1000 mg and (**B**) 100/1000 mg. Data are presented as mean ± standard deviation (SD) (*n* = 12).

**Figure 2:**
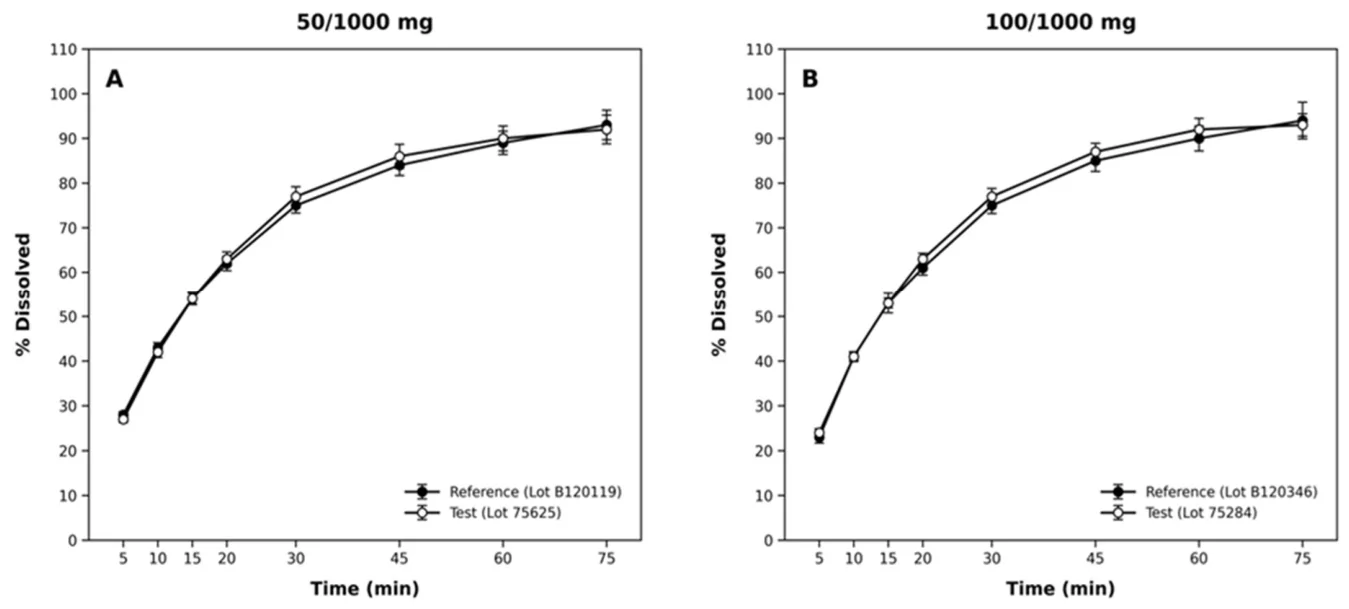
Mean comparative dissolution profiles of metformin from test (white circles) and reference (black circles) sitagliptin/metformin extended-release fixed-dose combination tablets. The panels represent: (**A**) 50/1000 mg and (**B**) 100/1000 mg. Data are presented as mean ± standard deviation (SD) (*n* = 12).

**Figure 3:**
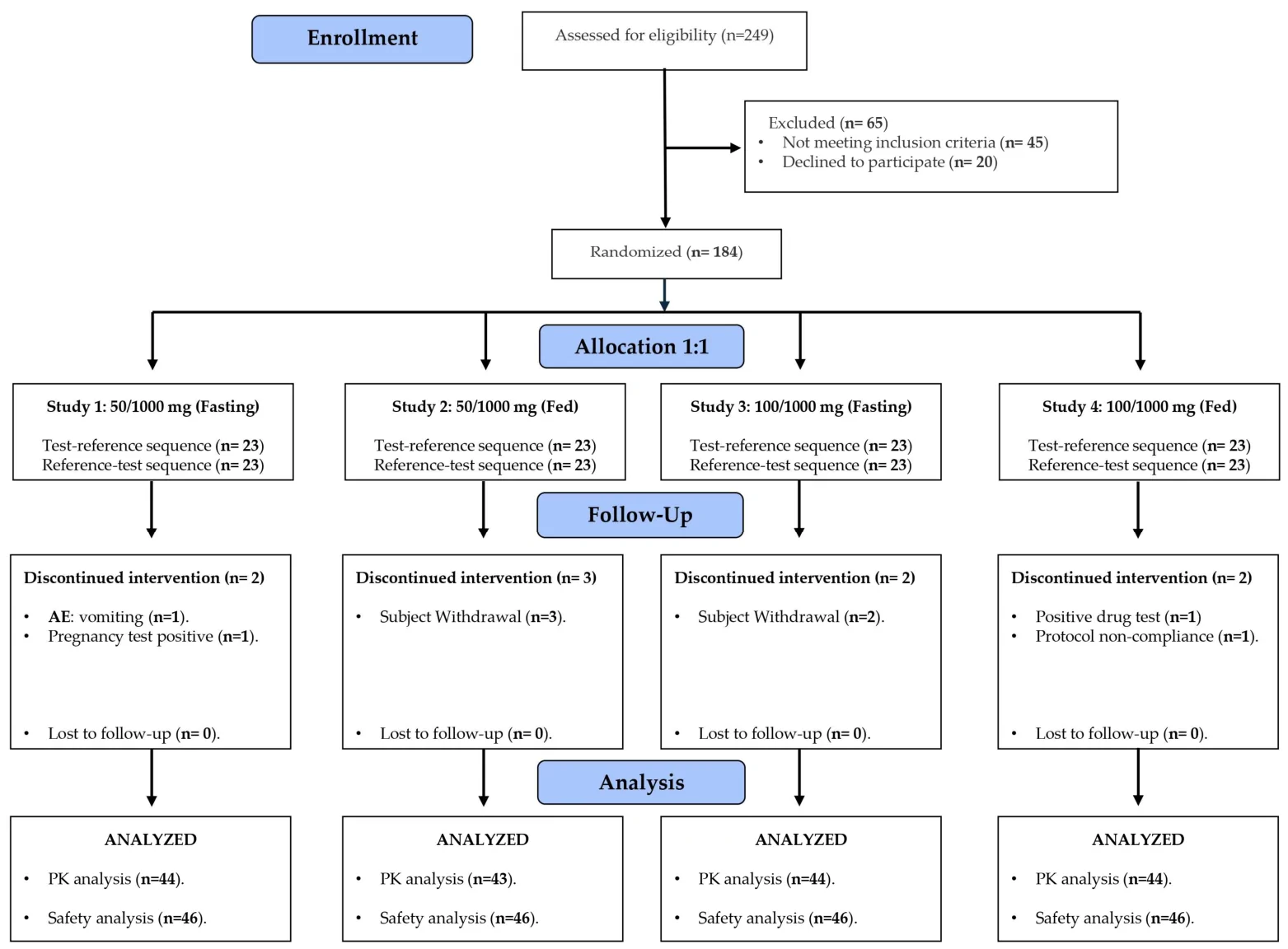
Study flowchart. *n*: number of subjects; PK: pharmacokinetics.

**Figure 4:**
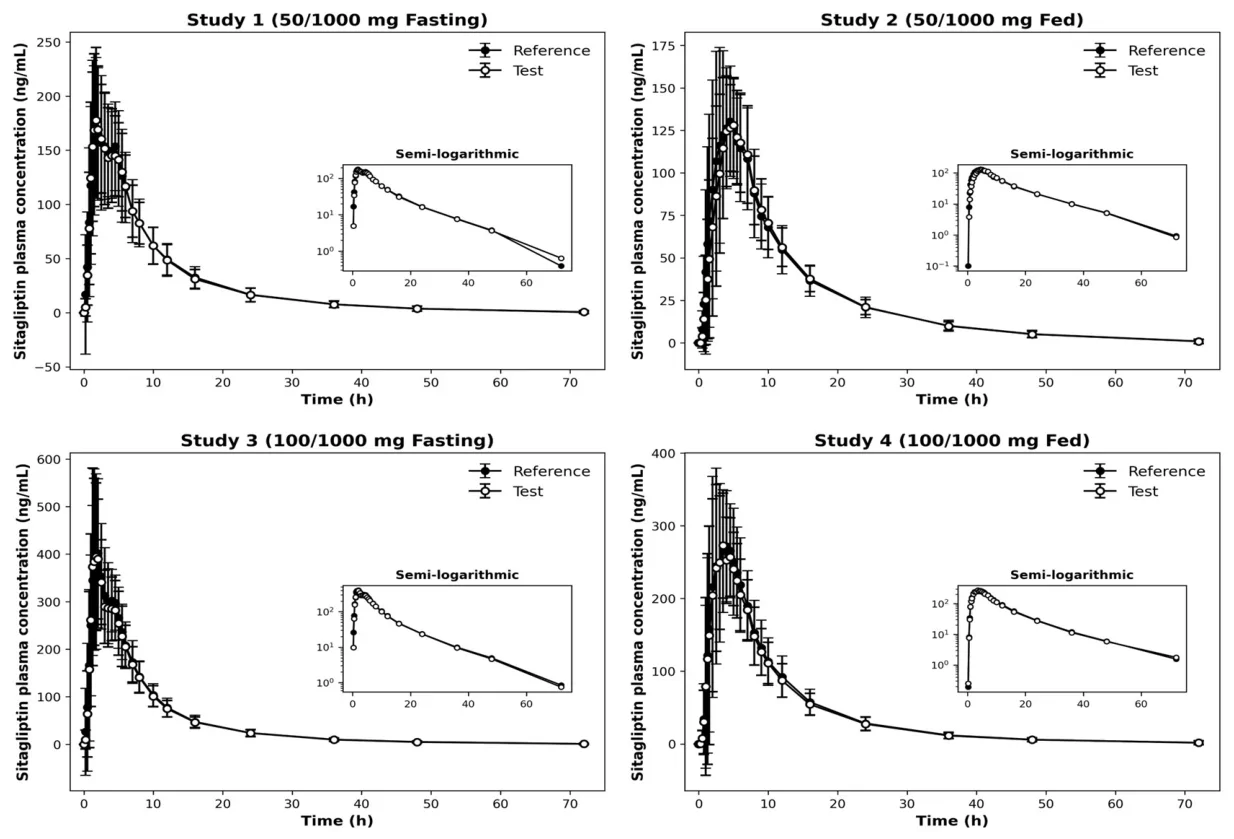
Mean plasma concentration–time profiles of sitagliptin following a single oral administration of the test (solid black line with open circles) and reference (solid black line with closed circles) sitagliptin/metformin extended-release (ER) fixed-dose combinations. The panels represent the evaluated cohorts: Study 1 (50/1000 mg, fasting conditions), Study 2 (50/1000 mg, fed conditions), Study 3 (100/1000 mg, fasting conditions), and Study 4 (100/1000 mg, fed conditions). Data are expressed as the mean ± standard deviation (SD). Insets show the corresponding semi-logarithmic profiles without SD bars for visual clarity.

**Figure 5:**
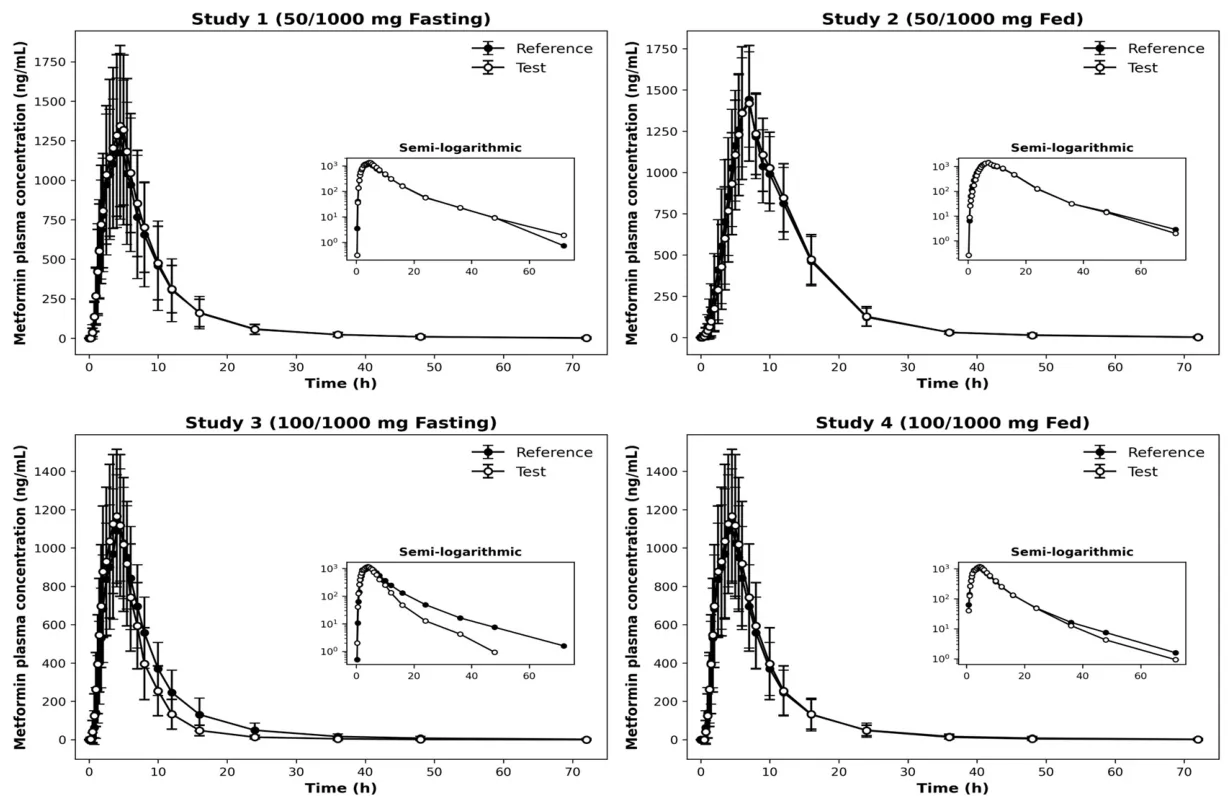
Mean plasma concentration–time profiles of metformin following a single oral administration of the test (solid black line with open circles) and reference (solid black line with closed circles) sitagliptin/metformin extended-release (ER) fixed-dose combinations. The panels represent the evaluated cohorts: Study 1 (50/1000 mg, fasting conditions), Study 2 (50/1000 mg, fed conditions), Study 3 (100/1000 mg, fasting conditions), and Study 4 (100/1000 mg, fed conditions). Data are expressed as the mean ± standard deviation (SD). Insets show the corresponding semi-logarithmic profiles without SD bars for visual clarity.

**Table 1:** Summary of baseline demographic characteristics of the healthy Mexican volunteers enrolled in the fasting and fed bioequivalence studies.

Variable	Study 1: 50/1000 mg (Fasting)	Study 2: 50/1000 mg (Fed)	Study 3: 100/1000 mg (Fasting)	Study 4: 100/1000 mg (Fed)
Enrolled (*n*)	46	46	46	46
Gender, *n* (%)				
Male	18	37	24	19
Female	28	9	22	27
Age (years) Mean ± SD	37 ± 12	39 ± 10	38 ± 10	34 ± 10
Weight (kg) Mean ± SD	62.2 ± 7.7	69.3 ± 10.9	62.7 ± 8.08	62.9 ± 9.6
Height (m) Mean ± SD	1.63 ± 0.07	1.68 ± 0.10	1.63 ± 0.08	1.62 ± 0.09
BMI (kg/m^2^) Mean ± SD	23.4 ± 2.0	24.3 ± 2.2	23.5 ± 2.36	23.7 ± 2.5

SD: standard deviation; *n*: number of subjects; BMI: body mass index; kg: kilograms; m: meters.

**Table 2:** Pharmacokinetic parameters of sitagliptin and metformin extended-release tablets following a single oral dose in healthy Mexican volunteers under fasting and fed conditions. Data are presented as mean ± standard deviation (SD).

PK Parameter	Sitagliptin (Test)	Sitagliptin (Reference)	Metformin (Test)	Metformin (Reference)
Study 1: 50/1000 mg (Fasting)				
C_max_ (ng/mL)	212.02 ± 64.79	220.13 ± 64.04	1502.32 ± 535.35	1393.09 ± 526.99
t_max_ (h)	2.27 ± 1.29	2.37 ± 1.18	4.35 ± 0.83	4.12 ± 1.20
AUC_0–t_ (ng·h/mL)	1807.96 ± 448.25	1828.59 ± 480.58	11,492.68 ± 4495.00	10,859.14 ± 4682.63
AUC_0-∞_ (ng·h/mL)	1874.41 ± 463.20	1892.09 ± 494.33	11,720.98 ± 4535.41	11,082.95 ± 4727.63
t_1/2_ (h)	11.42 ± 3.25	11.51 ± 2.93	8.86 ± 7.24	8.32 ± 6.48
Study 2: 50/1000 mg (Fed)				
C_max_ (ng/mL)	146.85 ± 31.19	158.40 ± 42.08	1476.95 ± 353.67	1502.90 ± 305.36
t_max_ (h)	4.48 ± 1.33	4.13 ± 1.45	6.83 ± 1.12	6.65 ± 0.78
AUC_0–t_ (ng·h/mL)	1732.30 ± 301.08	1746.78 ± 327.66	16,242.31 ± 3233.52	16,366.96 ± 2732.09
AUC_0-∞_ (ng·h/mL)	1809.57 ± 299.67	1833.36 ± 316.53	16,458.46 ± 3251.64	16,648.95 ± 2721.54
t_1/2_ (h)	12.35 ± 2.91	12.54 ± 3.76	7.46 ± 5.76	9.53 ± 12.01
Study 3: 100/1000 mg (Fasting)				
C_max_ (ng/mL)	506.26 ± 160.96	512.07 ± 169.64	1343.41 ± 393.74	1231.19 ± 281.16
t_max_ (h)	1.93 ± 0.96	1.98 ± 1.01	4.31 ± 0.90	4.25 ± 0.92
AUC_0–t_ (ng·h/mL)	3184.81 ± 658.07	3279.01 ± 684.06	9481.38 ± 2953.27	9177.96 ± 2544.11
AUC_0-∞_ (ng·h/mL)	3242.56 ± 580.64	3344.24 ± 690.44	9719.39 ± 2956.70	9394.07 ± 2572.19
t_1/2_ (h)	10.91 ± 3.05	11.72 ± 4.45	9.00± 10.14	8.50 ± 7.85
Study 4: 100/1000 mg (Fed)				
C_max_ (ng/mL)	336.71 ± 77.01	348.69 ± 94.29	1121.88 ± 219.10	1113.41 ± 206.35
t_max_ (h)	3.29 ± 1.33	3.39 ± 1.49	6.50 ± 1.12	6.50 ± 0.68
AUC_0–t_ (ng·h/mL)	2960.85 ± 465.36	3059.65 ± 604.81	12,573.86 ± 2904.56	12,544.26 ± 2941.53
AUC_0-∞_ (ng·h/mL)	3038.22 ± 483.97	3139.20 ± 618.99	12,750.57 ± 2916.38	12,756.92 ± 2923.05
t_1/2_ (h)	12.27 ± 6.59	11.74 ± 4.97	6.38 ± 2.33	8.25 ± 10.71

C_max_: maximum observed plasma concentration; t_max_: time to reach maximum plasma concentration; AUC_0–t_: area under the plasma concentration–time curve from time zero to the last quantifiable concentration; AUC_0-∞_: area under the plasma concentration–time curve from time zero extrapolated to infinity; t_1/2_: terminal elimination half-life.

**Table 3:** Geometric mean ratios, 90% confidence intervals (CIs), intra-subject coefficients of variation, and statistical power for the primary pharmacokinetic parameters.

Study/Analyte	Parameter	GMR (%)	90% CI (LL–UL)	CVintra (%)	Power
Study 1: 50/1000 mg (Fasting)					
Sitagliptin	C_max_	96.11	89.72–102.96	19.4	1.00
	AUC_0–t_	99.26	96.06–102.58	9.2	1.00
	AUC_0-∞_	99.43	96.47–102.49	8.5	1.00
Metformin	C_max_	108.76	101.93–116.05	18.2	0.97
	AUC_0–t_	106.91	101.33–112.80	15.0	1.00
	AUC_0-∞_	106.81	101.24–112.70	15.0	1.00
Study 2: 50/1000 mg (Fed)					
Sitagliptin	C_max_	93.70	89.35–98.26	13.1	1.00
	AUC_0–t_	99.35	96.31–102.49	8.6	1.00
	AUC_0-∞_	98.70	96.21–101.26	7.0	1.00
Metformin	C_max_	97.90	94.09–101.86	10.9	1.00
	AUC_0–t_	98.43	94.92–102.07	10.0	1.00
	AUC_0-∞_	98.03	94.63–101.56	9.7	1.00
Study 3: 100/1000 mg (Fasting)					
Sitagliptin	C_max_	98.82	91.50–106.72	21.7	1.00
	AUC_0–t_	97.48	94.27–100.81	9.4	1.00
	AUC_0-∞_	97.33	94.13–100.64	9.3	1.00
Metformin	C_max_	107.28	100.82–114.15	17.4	0.99
	AUC_0–t_	102.07	96.32–108.17	16.3	1.00
	AUC_0-∞_	102.36	96.62–108.44	16.2	1.00
Study 4: 100/1000 mg (Fed)					
Sitagliptin	C_max_	97.82	91.25–104.86	19.5	1.00
	AUC_0–t_	97.61	95.04–100.26	7.5	1.00
	AUC_0-∞_	97.59	94.95–100.31	7.7	1.00
Metformin	C_max_	100.64	97.14–104.27	9.9	1.00
	AUC_0–t_	100.67	97.84–103.59	8.0	1.00
	AUC_0-∞_	100.33	97.71–103.03	7.4	1.00

**CV_intra_:** Within-subject coefficient of variation; **GMR**: Geometric Mean Ratio. **LL:** Lower Limit; **UL:** Upper Limit. **C_max_:** maximum observed plasma concentration; **AUC_0–t_:** area under the plasma concentration–time curve from time zero to the last quantifiable concentration. **AUC_0–∞_:** area under the plasma concentration–time curve from time zero extrapolated to infinity.

**Table 4:** Summary of adverse events (AEs) by study and treatment.

Study Condition	Adverse Event	Test Group AEs	Reference Group AEs	Total AEs
Fasting Studies	Headache	5	4	9
	Dyspepsia	1	0	1
	Vomiting	1	0	1
Fasting Subtotal		7	4	11
Fed Studies	Headache	11	4	15
	Dyspepsia	1	1	2
	Loose stools	2	1	3
	Muscle contracture	1	0	1
	Nausea	0	1	1
Fed Subtotal		15	7	22
Total		22	11	33

## Data Availability

The original contributions presented in this study are included in the article/Supplementary Materials. Further inquiries can be directed to the corresponding authors.

## Supplementary Materials

The following supporting information can be downloaded at: https://www.mdpi.com/article/10.3390/ph19101532/s1, Table S1: Measured concentrations and variability across three independent calibration runs for the evaluation of method linearity of Sitagliptin in human plasma. Table S2: Calibration curve parameters for Sitagliptin. Table S3: Measured concentrations and variability across three independent calibration runs for the evaluation of method linearity of Metformin in human plasma. Table S4: Calibration curve parameters for Metformin. Figure S1: Representative calibration curve obtained using analyte-to-internal standard peak area ratios, showing the linear relationship between concentration and response for Sitagliptin in human plasma. Figure S2: Representative calibration curve obtained using analyte-to-internal standard peak area ratios, showing the linear relationship between concentration and response for Metformin in human plasma. Table S5: Summary of bioanalytical method validation results for Sitagliptin and Metformin.

## Abbreviations

The following abbreviations are used in this manuscript:

AEs	Adverse events
ANOVA	Analysis of variance
API	Active pharmaceutical ingredient
AUC_0–t_	Area under the plasma concentration–time curve from time zero to the last quantifiable concentration
AUC_0–∞_	Area under the plasma concentration–time curve from time zero extrapolated to infinity
BCS	Biopharmaceutics Classification System
BMI	Body mass index
CEI	Ethics in Research Committee (Comité de Ética en Investigación)
CI	Confidence interval
C_max_	Maximum observed plasma concentration
COFEPRIS	Federal Commission for the Protection against Sanitary Risks (Comisión Federal para la Protección contra Riesgos Sanitarios)
CV_intra_	Intra-subject coefficient of variation
CYP	Cytochrome P450
DPP-4	Dipeptidyl peptidase-4
ECG	Electrocardiogram
ER	Extended-release
ESI^+^	Positive electrospray ionization mode
f_2_	Similarity factor
FDA	Food and Drug Administration
FDC	Fixed-dose combination
GCP	Good Clinical Practice
GIP	Glucose-dependent insulinotropic polypeptide
GLM	Generalized Linear Model
GLP	Good Laboratory Practice
GLP-1	Glucagon-like peptide-1
GMR	Geometric mean ratio
HIV	Human Immunodeficiency Virus
hOAT-3	Human organic anion transporter-3
HPLC-UV	High-performance liquid chromatography coupled with UV detection
ICH	International Council for Harmonization
IR	Immediate-release
IRC	Institutional Research Committee
ITT	Intention-to-treat
IVIVC	In vitro–in vivo correlation
K_2_EDTA	Dipotassium ethylenediaminetetraacetic acid
Ke	Terminal elimination rate constant
LC-MS/MS	Liquid chromatography–tandem mass spectrometry
LL	Lower limit
MRM	Multiple reaction monitoring
MRT	Mean residence time
NCA	Non-compartmental analysis
OCT1	Organic cation transporter 1
P-gp	P-glycoprotein
PK	Pharmacokinetic
PMAT	Plasma membrane monoamine transporter
PP	Per-protocol
QC	Quality control
SAEs	Serious adverse events
SD	Standard deviation
SPE	Solid-phase extraction
T2D	Type 2 Diabetes
t_1/2_	Terminal elimination half-life
t_max_	Time to reach peak plasma concentration
TOST	Two one-sided tests
UL	Upper limit
VDRL	Venereal Disease Research Laboratory
