# Supplementary material for: Bioequivalence Assessment of Sitagliptin/Metformin Extended-Release Fixed-Dose Combinations in Healthy Mexican Subjects Under Fasting and Fed Conditions
Source: Pharmaceuticals (Basel). 2026 Sep 27;19(10):1532. doi: 10.3390/ph19101532 (PMC13708307; doi:10.3390/ph19101532)
Supplement: Supplementary file 1 [file pharmaceuticals-19-01532-s001.zip › pharmaceuticals-4545577-supplementary.pdf]

1. Supplementary material: Bioanalytical Method Validation

Validation of an Analytical Method for the Simultaneous Determination of Sitagliptin and Metformin in Human K_2EDTA Plasma by LC–MS/MS (Information extracted and summarized from the bioanalytical validation report)

1.1 Objective and regulatory framework

The aim of this study was the full validation of an analytical method for the simultaneous determination of Sitagliptin and Metformin in human K_2EDTA plasma.

The bioanalytical method validation was conducted in accordance with the applicable regulatory requirements for bioequivalence studies in Mexico (NOM-177-SSA1-2013). The validation approach is consistent with internationally accepted principles described in regulatory guidelines, including those from the European Medicines Agency (EMA) and the U.S. Food and Drug Administration (FDA), as well as Good Laboratory Practice (GLP) standards.

1.2 Summary of the analytical method

Sitagliptin and Metformin concentrations in human K_2EDTA plasma were determined using a validated LC-MS/MS method with Sitagliptin-d4 and Metformin-d6 as internal standards (IS). Sample preparation was performed by solid-phase extraction (SPE). Quantification was based on analyte-to-internal standard peak area ratios, and calibration curves were constructed using least-squares linear regression with a $1/x^2$ weighting factor. The same validated LC–MS/MS method was used for the quantification of Sitagliptin and Metformin plasma concentrations in both bioequivalence studies evaluated in this work.

1.2.1 Linearity and calibration curve

Calibration standards were prepared in human plasma over the concentration ranges of 2.00–600.21 ng/mL for Sitagliptin and 10.0–3001.0 ng/mL for Metformin. Calibration curves were constructed using analyte-to-internal standard peak area ratios across the studied concentration range.

The calibration model showed excellent linearity, with mean correlation coefficient (r) of 0.99960 for Sitagliptin and 0.99964 for Metformin.

Table S1. Measured concentrations and variability across three independent calibration runs for the evaluation of method linearity of Sitagliptin in human plasma.

Run	**Nominal concentration** (ng/mL)							
	**2.00**	**4.00**	**10.00**	**65.54**	**131.09**	**262.17**	**468.16**	**600.21**
1	2.00	4.02	9.85	64.18	138.20	255.51	453.70	619.42
2	1.99	4.15	9.44	64.33	129.76	262.56	483.91	612.07
3	1.95	4.20	9.96	68.26	127.59	254.55	459.57	607.07
**Mean**	1.980	4.123	9.750	65.590	131.850	257.540	465.727	612.853
**SD**	0.0265	0.0929	0.2740	2.3135	5.6053	4.3739	16.0184	6.2122
**%CV**	1.3	2.3	2.8	3.5	4.3	1.7	3.4	1.0
**%Abs. Dev.**	1.0	3.1	2.5	0.1	0.6	1.8	0.5	2.1

SD: standard deviation; %CV: coefficient of variation (%), %Abs Dev: absolute deviation.

Table S2. Calibration curve parameters for Sitagliptin.

Run	Slope (m)	Intercept (b)	Correlation coefficient (r)
1	1.0084	-1.1642	0.9991
2	1.0255	-1.7978	0.9999
3	0.9993	-1.1233	0.9997
**Mean**	1.01111	-1.36176	0.99960

Table S3. Measured concentrations and variability across three independent calibration runs for the evaluation of method linearity of Metformin in human plasma.

Run	**Nominal concentration** (ng/mL)							
	**10.00**	**20.00**	**50.0**	**292.6**	**585.2**	**1170.4**	**2340.8**	**3001.0**
1	10.1	19.2	53.6	275.3	605.0	1129.8	2285.8	3128.5
2	10.1	19.8	47.6	296.6	596.2	1174.2	2365.6	2999.7
3	10.0	20.5	47.7	297.5	579.3	1184.6	2319.9	3047.4
**Mean**	10.07	19.83	49.63	289.80	593.50	1162.87	2323.77	3058.53
**SD**	0.058	0.651	3.436	12.565	13.061	29.105	40.040	65.118
**%CV**	0.6	3.3	6.9	4.3	2.2	2.5	1.7	2.1
**%Abs. Dev.**	0.7	0.8	0.7	1.0	1.4	0.6	0.7	1.9

SD: standard deviation; %CV: coefficient of variation (%), %Abs Dev: absolute deviation.

Table S4. Calibration curve parameters for Metformin.

Run	Slope (m)	Intercept (b)	Correlation coefficient (r)
1	1.0191	-13.2106	0.9991
2	1.0031	2.0749	1.0000
3	1.0076	-2.4438	0.9999
**Mean**	1.00993	-4.52651	0.99964

Figure S1. Representative calibration curve obtained using analyte-to-internal standard peak area ratios, showing the linear relationship between concentration and response for Sitagliptin in human plasma.

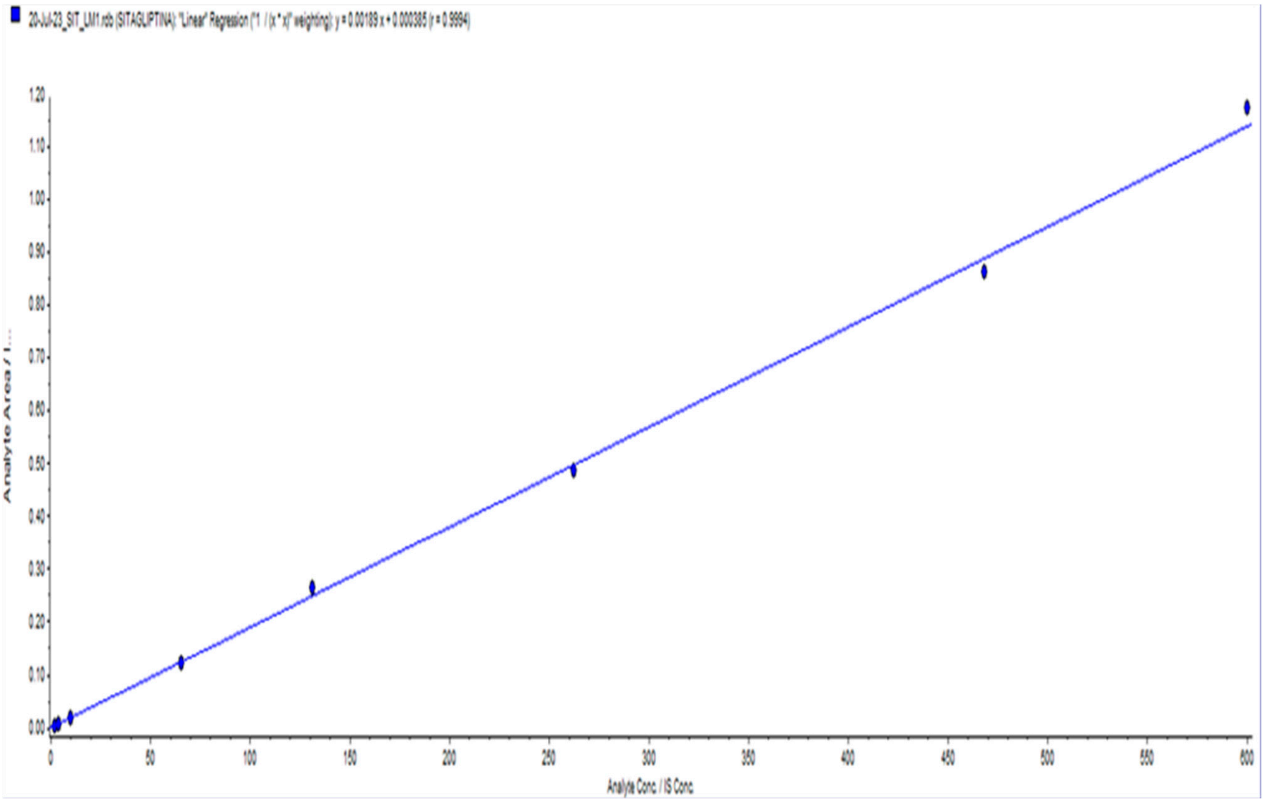

Figure S2. Representative calibration curve obtained using analyte-to-internal standard peak area ratios, showing the linear relationship between concentration and response for Metformin in human plasma.

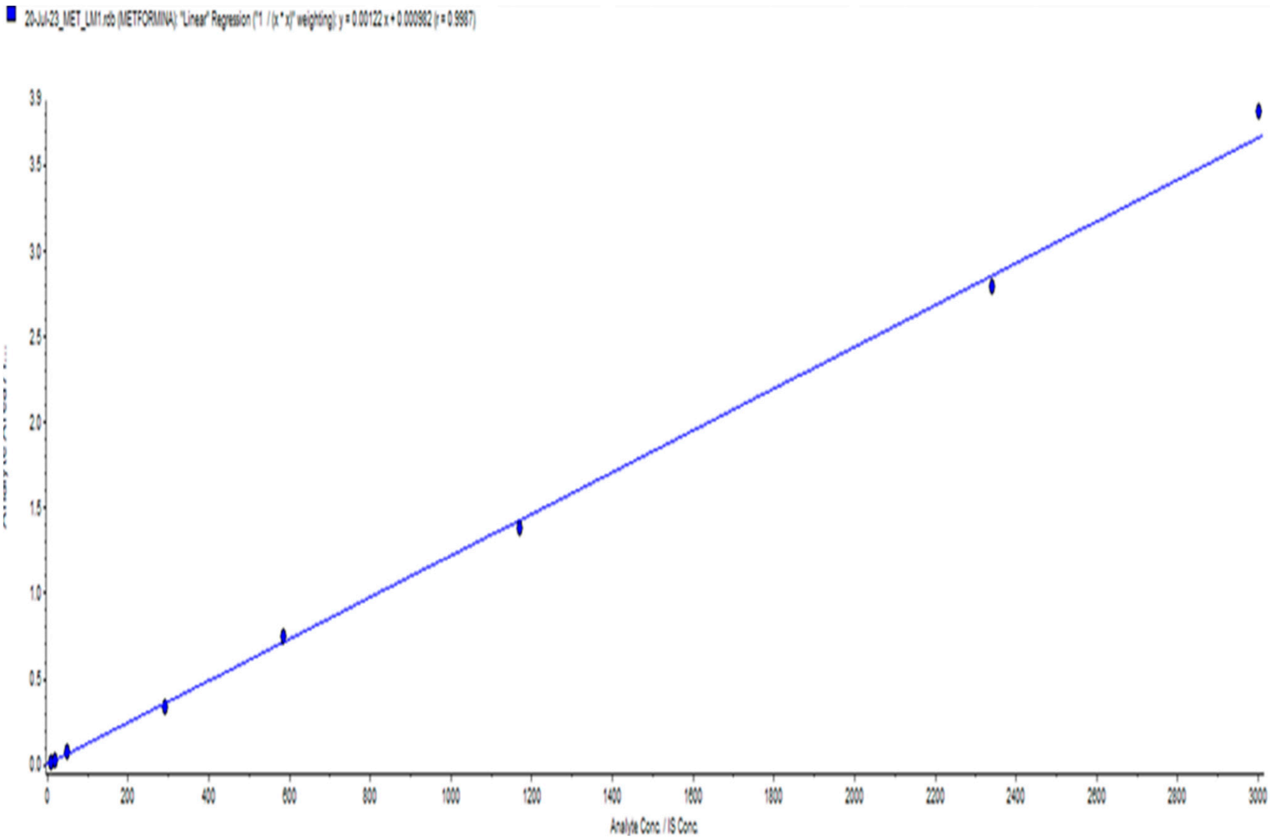

1.2.2 Accuracy and precision

Intra-day and inter-day accuracy and precision were evaluated for both Sitagliptin and Metformin using quality control (QC) samples at multiple concentration levels.

The method showed adequate precision, with coefficients of variation (CV) below 15%, and accuracy expressed as absolute deviation (AD) within 15% of nominal concentrations across all QC (quality control) levels for both analytes. For the lower limit of quantification (LLOQ), both precision (CV≤20%) and accuracy (AD ≤ 20%) complied with the predefined acceptance criteria.

1.2.3 Selectivity and matrix effect

Selectivity was evaluated using multiple individual plasma sources, including hemolyzed and lipemic samples, as well as plasma containing commonly co-administered drugs.

No significant interference was observed at the retention times of Sitagliptin, Metformin, and their corresponding internal standards . Matrix effects were assessed and demonstrated

acceptable variability, with normalized matrix factor CV values below 15% at both QC levels for both analytes.

1.2.4 Recovery

Extraction recovery was evaluated at different concentration levels. The method showed consistent recovery across the concentration range for both Sitagliptin and Metformin, as well as their corresponding internal standards, with CV values below 15% at all evaluated concentration levels.

1.2.5 Lower limit of quantification (LLOQ)

The LLOQ was established at 2.00 ng/mL for Sitagliptin and 10.00 ng/mL for Metformin, demonstrating acceptable accuracy and precision (CV ≤ 20% and absolute deviation ≤20%), thereby confirming the suitability of the method for quantifying low plasma concentrations of both analytes.

1.2.6 Stability

Stability of Empagliflozin and Metformin in plasma was evaluated under various conditions, including:

- Short-term (room temperature) stability.
- Autosampler stability.
- Freeze–thaw cycles.
- Long-term storage stability.
- Refrigerated processed sample stability.
- Working solution stability (short- and long-term).
- Stock solution stability (short- and long-term).
- Whole blood stability.

Both analytes were found to be stable under all tested conditions, with precision (CV ≤ 15% and accuracy expressed as absolute deviation ≤ 15%) within the predefined acceptance criteria.

1.3 Overall validation performance

The validation results demonstrated that the analytical method met all predefined acceptance criteria for bioanalytical method validation. The method showed adequate linearity, precision, accuracy expressed as absolute deviation, selectivity, recovery, sensitivity, and stability under all evaluated conditions for both Sitagliptin and Meftformin.

Table S5. Summary of bioanalytical method validation results for Sitagliptin and Metformin.

Parameter	Acceptance criteria	Result Sitagliptin	Result Metformin
Linearity	r ≥ 0.98	0.99960	0.99964
Precision (intra-day)	CV ≤ 15%	1.9-3.4%	2.1-3.6%
Accuracy (intra-day)	Absolute deviation (AD) ≤15%	1.2-9.4%	5.5-11.3%
Precision (inter-day)	CV ≤ 15%	3.3-4.7%	2.7-6.9%
Accuracy (inter-day)	AD ≤15%	1.3-3.5%	0.4-4.0%
Selectivity	No interference at the retention time of the analyte and internal standard	No interference was observed at the retention time of Sitagliptin and the IS in blank, hemolyzed, and lipemic plasma, or in the presence of tested drugs (acetaminophen, ketorolac, ondansetron, loratadine, salicylic acid, metamizole, and loperamide)	No interference was observed at the retention time of Metformin and the IS in blank, hemolyzed, and lipemic plasma, or in the presence of tested drugs (acetaminophen, ketorolac, ondansetron, loratadine, salicylic acid, metamizole, and loperamide)
LLOQ	CV ≤ 20%, AD ≤20%	(CV 8.2%, AD 3.4)	(CV 4.3%, AD 0.7)
Carryover	Carryover at the analyte retention time < 20% and carryover at the	0.0% for Sitagliptin and 0.0% for Sitagliptin -d4	0.0% for metformin and 0.2% metformin-d6

	internal standard (IS) retention time < 5%		
Recovery	CV ≤ 15%	9.9% (Sitalgiptin), 7.1 % (Sitalgiptin -d4)	9.3%(Metformin), 2.1 % (Metformin-d6)
Reinjection reproducibility	CV ≤ 15%, AD ≤ 15%	Stable up to 20.78 h (CV 2.1–6.7%, AD 2.4-3.9%)	Stable up to 20.78 h (CV 2.9-4.3%, AD 0.3–2.1%)
Matrix effect	Normalized matrix factor CV ≤ 15% at both QC levels	4.3% (low QC), 3.7% (high QC)	0.7% (low QC), 0.6% (high QC)
Stability (short-term, room temperature)	CV ≤ 15%, AD ≤ 15%	Stable up to 21.77 h (CV 1.2%-4.1%, AD 1.8%-3.3%)	Stable up to 22.57 h (CV 3.0%-5.0%, AD 2.0%-2.3%)
Stability (autosampler)	CV ≤ 15%, AD ≤ 15%	Stable up to 51.93 h (CV 2.7%-3.9%, AD 0.7-5.5%)	Stable up to 51.93 h (CV 2.7%-8.0%, AD 2.3%-3.6%)
Freeze–thaw stability (-70°C)	CV ≤ 15%, AD ≤ 15%	Stable for 8 cycles (CV 2.9%-4.5%, AD 1.0%-4.6%)	Stable for 8 cycles (CV 2.3%-2.4%, AD 1.7-3.5%)
Long-term stability	CV ≤ 15%, AD ≤ 15%	Stable up to 153 days (CV 0.6%-2.1%, AD 4.7-5.6%)	Stable up to 153 days (CV 0.5%-1.5%, AD 6.3-13.0%)
Refrigerated processed sample stability	CV ≤ 15%, AD ≤ 15%	Stable up to 48.02 h (CV 0.9-3.2%, AD 1.3-2.5%)	Stable up to 48.02 h (CV 1.1-4.1%, AD 3.0-2.6%)

Working solution stability (short- and long-term)	AD ≤ 10%	Stable up to 23.87 h (high, short-term), 24.03 h (low, short-term), and 154 days (high and low,long-term)<br><br>CV 1.6–1.7% and AD 1.6–2.4% (high, short-term); CV 2.0–4.9% and AD 1.0–3.5% (low, short-term); CV 0.3–0.5% and AD 2.2–2.8% (high, long-term); CV 1.0–2.1% and AD 1.7–2.2% (low, long-term)	Stable up to 23.87 h (high, short-term), 24.03 h (low, short-term), and 154 days (high and low,long-term)<br><br>CV 1.0–2.9% and AD 1.6-2.0% (high, short-term); CV 2.6-2.7% and AD 2.7–3.9% (low, short-term); CV 1.5-1.8% and AD 4.8–5.6% (high, long-term); CV 1.9–2.1% and AD 1.2–1.4% (low, long-term)
Stock solution stability (short- and long-term)	AD ≤ 10%	Stable up to 23.85 h (short-term) and 154 days (long-term); CV 1.8–1.8% and AD 0.5-0.7% (short-term); CV 1.0–1.5% and AD 5.0-9.6% (long-term)	Stable up to 23.85 h (short-term) and 154 days (long-term); CV 0.9–2.7% and AD 0.3–2.2% (short-term); CV 1.2–1.6% and AD 7.5–8.3% (long-term)
Whole blood stability	CV ≤ 15%, AD ≤ 15%	Stable up to 3.02 h (CV 3.4-3.4%, AD 0.3%-0.5%)	Stable up to 3.02 h (CV 5.0-5.1%, AD 2.2–4.1%)
